# Challenges and strategies for cohort retention and data collection in an indigenous population: Australian Aboriginal Birth Cohort

**DOI:** 10.1186/1471-2288-14-31

**Published:** 2014-02-26

**Authors:** Megan Lawrance, Susan M Sayers, Gurmeet R Singh

**Affiliations:** 1 C/-Menzies School of Health Research, Institute of Advanced Studies, Charles Darwin University, PO Box 41096, Casuarina, NT 0810, Australia; 2 Charles Darwin University & Flinders | School of Medicine Flinders University, PO Box 41096, Casuarina, NT 0811, Australia

**Keywords:** Epidemiological method, Prospective longitudinal cohort, Ethnic minority, Health determinants, Australian, Aboriginal

## Abstract

**Background:**

Longitudinal prospective birth cohort studies are pivotal to identifying fundamental causes and determinants of disease and health over the life course. There is limited information about the challenges, retention, and collection strategies in the study of Indigenous populations. The aim is to describe the follow-up rates of an Australian Aboriginal Birth Cohort study and how they were achieved.

**Methods:**

Participants were 686 babies enrolled between January 1987 and March 1990, born to a mother recorded in the Delivery Suite Register of the Royal Darwin Hospital (RDH) as a self-identified Aboriginal. The majority of the participants (70%) resided in Northern Territory within rural, remote and very remote Aboriginal communities that maintain traditional connections to their land and culture. The Aboriginal communities are within a sparsely populated (0.2 people/ km2) area of approximately 900,000 km2 (347sq miles), with poor communication and transport infrastructures. Follow-ups collecting biomedical and lifestyle data directly from participants in over 40 locations were conducted at 11.4 years (Wave-2) and 18.2 years (Wave-3), with Wave-4 follow-up currently underway.

**Results:**

Follow-ups at 11 and 18 years of age successfully examined 86% and 72% of living participants respectively. Strategies addressing logistic, cultural and ethical challenges are documented.

**Conclusions:**

Satisfactory follow-up rates of a prospective longitudinal Indigenous birth cohort with traditional characteristics are possible while maintaining scientific rigor in a challenging setting. Approaches included flexibility, respect, and transparent communication along with the adoption of culturally sensitive behaviours. This work should inform and assist researchers undertaking or planning similar studies in Indigenous and developing populations.

## Background

A prospective longitudinal study of a birth cohort is a well recognized way of identifying the temporal causes and determinants of disease and health over the life course. As a result, birth cohort studies are being maintained for many years, including some that are now in their seventh decade [1,2], and new birth cohorts are being established [3-6]. International collaborations of cohort studies are also being encouraged, particularly in societies undergoing economic and demographic transitions [7]. Despite these initiatives, there is limited information about the life course of disease in Indigenous and developing populations, even though these populations contribute disproportionably to the burden of non-communicable disease world-wide, namely high rates of diabetes and cardiovascular heart disease [8].

In the Northern Territory (NT) of Australia, the Aboriginal population has concurrently high rates of low birth weight, infant under-nutrition and infection, as well as adult diabetes, renal disease and cardiovascular disease, all contributing to a disproportionately high rate of premature adult death.

In 1987 a prospective longitudinal study of an Australian Aboriginal Birth Cohort (ABC) was established with a focus on the development of chronic non-communicable diseases [9]. Two follow-ups have been conducted, and the third follow-up is currently underway. While this cohort study presents retention challenges that are common to conventional longitudinal cohort studies, other challenges have been identified. These challenges reflect the geographic and cultural environment of the Indigenous population being studied. This work will help to inform and assist researchers undertaking or planning similar studies in Indigenous and developing populations. We aim to report the follow-up rates of an Aboriginal Birth Cohort study and describe how they were achieved.

### Participants

Babies were eligible for recruitment into the ABC if they were singletons born January 1987- March 1990, to a mother recorded in the Delivery Suite Register of the Royal Darwin Hospital (RDH) as a self-identified Aboriginal. Of the 1253 babies eligible for recruitment, 686 were enrolled into the study (Wave-1). Recruitment was dependent on the availability of the recruiting paediatrician and although not randomly selected there was no significant differences in mean birth weight, sex ratio or the birth weight frequencies between those recruited and those not recruited [9]. Health care policy was for all women to deliver in hospital so women were relocated to the nearest hospital at 38 weeks or earlier if the delivery was deemed to be high risk. Hence the majority of the participants (70%) came from rural, remote and very remote Aboriginal communities which maintain traditional connections to the land and their culture.

### Setting

The RDH is the centre of health delivery for a large region of northern Australia. The Aboriginal communities are situated within this sparsely populated (0.2 people/km^2^) area of approximately 900,000 km^2^ (347sq miles) with poor communication and transport infrastructures. This catchment area is similar to the combined area of California and New México, with a population density less than Alaska. Although urban rural disparities occur, the rural areas have a well established network of government health services.

## Methods

Details of this prospective longitudinal study have been previously published [9,10]. In brief, follow-ups have been conducted at 11.4 years (Wave-2) and 18.2 years (Wave-3) with the current Wave-4 follow-up underway at approximately 26 years. Growth and nutritional measures, biomedical specimens and lifestyle questionnaires are directly collected from the participants at the point of contact.

### Ethics

This study is conducted within the principles of the National Health Medical Research Council Road Map 11 which is a strategic framework for improving the health of Aboriginal and Torres Strait Islander people through research [11]. It adheres to the themes of respect, integrity, responsiveness, competency, and reciprocity, common to the ethical guidelines for research involving Indigenous peoples in Australia, Canada and New Zealand [12-14].

The study has approval from The Human Research Ethics Committee of the NT Department of Health and Families, and Menzies School of Health Research, including an Aboriginal Ethical Sub-committee which has the power of veto. Written consent was obtained in the form of an itemized consent, giving the participant capacity to refuse individual procedures (Additional file 1).

## Results

### Follow up

**Figure 1:**
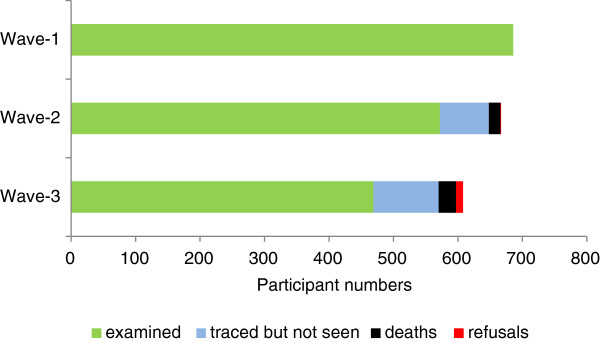
Aboriginal Birth Cohort: Participant outcomes for follow-up waves.

Twenty years after recruitment, vital status was determined for 87% of the cohort. Figure 1 shows that 86% and 72% of living participants were directly examined at mean ages 11.4 years (Wave -2) and 18.2 years (Wave-3).

Ninety three percent (93%) and 83% of participants were traced at 11.4 and 18.2 years respectively. Traced participants not examined (78, Wave -2 and 102, Wave-3) were due to refusals, the costs of examining individuals in very remote locations and delays of planned trips due to cyclone warnings, unexpected community events or poor flying conditions. Of the initial cohort, 20 years after recruitment only 91 participants could not be located.

### Challenges and responding strategies

The challenges of maintaining, tracing and undertaking data collection in this Indigenous cohort are predominantly of a logistical and cultural/linguistic nature.

For **maintenance** between follow-up waves, challenges related to the limited availability of good communication routes, with no television coverage and limited radio and newspapers in which to advertise and publicise the study. Personal participant contact is difficult between follow-ups as there are no individual household postal services, nor standard mobile phone coverage in remote areas. In larger communities there are fixed phone lines for the community council, school and health clinic, but in smaller communities phone contact is limited to a single solar powered phone, often not in working order.

There are cross-cultural differences between the researchers and the cohort participants as well as marked cultural diversity among the Aboriginal people within the cohort itself: Discreet communities may contain multiple land-owning clans, and this diversity is reflected in approximately 30 different dialects within the cohort region. As a result 70% of participants use English as a second language and in some cases, a third language.

Between waves, in order to develop confidence and familiarity with the project and the research team, we sought to establish the identity and legitimacy of the project and the research team members. In place of one to one contact, we fostered community relationships which helped identify key community members with local knowledge.

Key community people were important in all phases of the follow-up. They acted as project advocates, interpreters, helped with practical local navigation issues, facilitated community consultation and negotiation, and in some cases were employed as paid assistants (Table 1).

**Table 1:** Aboriginal Birth Cohort: strategies used to address maintenance challenges

**Aim**	**Responding strategies**
To establish project legitimacy and identity	Study tag, “Clan Cohort”, logo and ID cards developed
	Regular updates in local newsletters for Aboriginal child and family wellbeing services
	Articles published in Aboriginal and Torres Strait Islander Health Worker Journal
	Discussions on Indigenous radio stations
	Institutional Indigenous reference group presentations for consultation and negotiation
	Opportunistic informal discussion with Aboriginal Health Workers attending workshops and conferences in city
	Bright cartoon posters with simple English posted around communities with story of the study, its findings and overall long term objectives contributing to developing a sense of history
To establish researchers profiles	Continuity of the research team; cohort founder and recruiter still engaged with study, two other senior researchers for 12 years
	Researchers photos attached to leaflets and posters
	Cartoon posters with recognizable caricatures of the researchers posted around communities
To develop community relationships	Developing community relationships with Elders, Aboriginal councils and community health clinics, through phone, mail and personal meetings
	Attending community events, art shows, open days and festivals
	Sending Christmas cards, thank you notes and study updates with pictures and diagrams to community councils and clinics
	Use of photo albums from current and previous follow-ups
	At end of community visit sending summary of de-identified community health findings to Elders, council and health clinic
To establish researcher participant relationships	Cohort reference group presentations for advice on all aspects of study
	Cohort participants invited to “Researcher Thank You Day “with media involved
	Cartoon posters with simple English posted around communities
	Study aids with large non-verbal visual component accompanied by written information sheets
	Biomedical results in visual form given to cohort participants
	Cross-cultural training provided to researchers
	Limited field staff turnover

Photos of participants seen in the past were especially effective in fostering positive relationships and aiding in tracing. They provided concrete evidence that the researchers were known to the community, and participants reported enjoying seeing themselves, family members and peers in a hard copy album.

**Figure 2:**
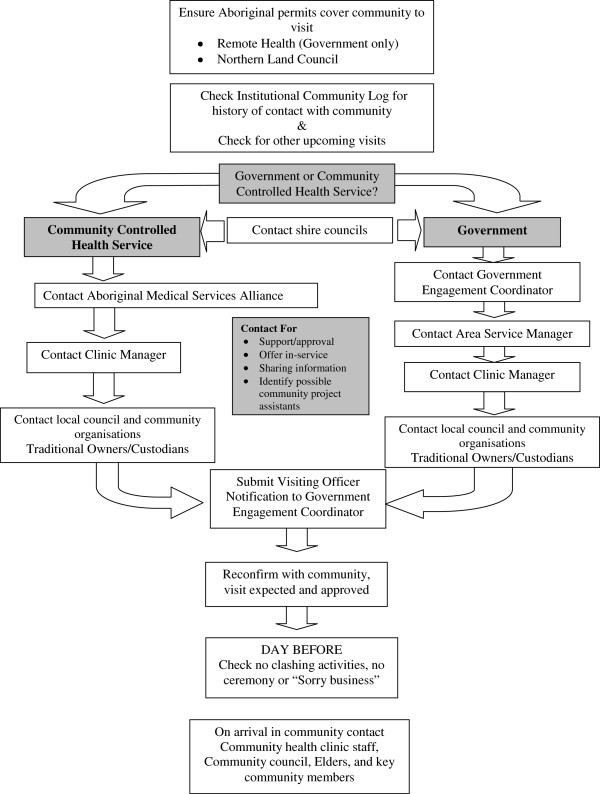
Aboriginal birth cohort: Community visit flow chart.

The procedures for **locating participants** have previously been described [10]. Briefly a multiphase method was used with separate arms for rural and urban participants. The locating phase was affected by the poor communication and linguistic challenges of the maintenance phase. While only a few urban participants had individual post boxes and even fewer responded to an introductory letter, the letter did serve as an ice breaker when face to face contact was made later. For rural visits, a generic community visit flow chart served as check list for staff organizing a visit see Figure 2.

Name changes and mobility also complicated localizing participants. When checked at the age of four years, 30% of participants had changed their name from the recruitment name. Participant mobility occurs within areas defined by kinship ties and within four years 18% had changed their place of residence since recruitment [9]. Importantly, family links between specific communities were identified (Table 2).

**Table 2:** Aboriginal Birth Cohort: strategies used to address locating challenges

**Aim**	**Responding strategies**
To find participants	
Rural	Key significant people identified in each large community and employed
	List of cohort members thought to be in community sent to them requesting confirmation of participant presence
Urban	Door to door visits yielded best results
	Letter useful
To positively identify participants	Multiple personal identifiers including unique hospital number, name, sex, date of birth and community residence at time of birth
	Showing photograph to community members
	Recording of aliases on spread sheet
	Key Aboriginal community assistants employed with local knowledge of community movements
	Visits to remote communities with strong kinship ties scheduled close together
To use personal images	All participants sign individual consents for image photograph to be taken and for photograph to be used for promotion, publicity and tracing

**Data** relating to growth and nutritional measures, biomedical specimens and lifestyle questionnaires were directly collected from the participants. Logistic challenges related to transporting the researchers and their equipment to key remote community hubs. There were also challenges related to the methods of giving information to, and seeking consent from participants. Consent and information sheets evolved with the increasing age of the cohort and changing ethical considerations leading finally to substantial use of visual aids and a written information sheet in simple English accompanying a visually enhanced staged consent form (Additional file 1). In Wave-3, 10% of participants chose to selectively opt out of different assessments within the staged consent (Table 3).

**Table 3:** Aboriginal Birth Cohort : data collection, challenges and strategies

**Aim**	**Challenges**	**Responding strategies**
To gain access to participants	Rural	
	Vast, sparsely populated area remote communities	Priority of dry season community assessments
	Poor unsealed roads and runways	Self-sufficient four wheel drive travel, fuel, food, water and satellite phone
	3-4 months monsoon flooding of roads and airstrips	Researchers trained in four wheel driving
		Local research assistants help navigate unmarked roads to community
		Road travel supplemented by light aircraft charter or commercial travel to larger hubs
		A research assistant with pilots licence an advantage
	Urban	
	Failure to respond to letter	Door to door transport provided
	Difficulties getting to appointments	Central clinic used for assessment
To consult and negotiate with communities to gain approval and fit with community activities	Multiple and support required	Generic flow chart developed to be used for each community (Figure 2)
To organise researcher team and satisfy needs	Limited space	Small multi-skilled research team
	Limited food outlets	Personal breakfast and lunch food, team roster for night meals
To transport equipment	Space and weight restrictions	Light equipment e.g., hand held ultrasound and vitalograph
		Researchers’ personal possessions limited
	Travel unsealed rough roads	Robust equipment purchased and wrapped in bubble wrap
To have constant power supply	Power unreliable in communities	All equipment capable of running with battery power
To explain procedures	English second language, participants unfamiliar with scientific terms and procedures	Visual aids, pictures drawings and demonstrations
		Simple English assisted by employment of local Aboriginal
		assistant
		Explanations in groups, max 4 gender matched with researcher
To collect data	Participants shy and unfamiliar with procedures	Siblings, cousins and friends data collection scheduled together for procedures
	Growth and nutritional data considered first priority	Triage of data collection making sure primary growth and nutritional data always obtained
	Lack of private space	Researchers carry multiple sarong lengths to screen off private spaces
To do a venepuncture	Participants scared of procedure	Local anaesthetic cream used
		Observation of others consenting to be watched during procedure
To transport biological specimen to distance laboratory	Preserving blood and urine samples	Blood centrifuged at point of collection
		Serum separated and placed in specific testing tubes
		Specimens maintained at low temperature in cold storage boxes or fridges
		Transported to central laboratory on government planes from hub if delays expected
To gain informed consent	English second language, participants unfamiliar with scientific terms and procedures	Staged consent form (Additional file 1) accompanied by visual aids explained by gender matched research assistant
To avoid clashing with community activities to	Ceremonies and “sorry business” Other agency and government department visits	Day before a planned community visit check still appropriate to visit
		Flexibility of researchers to change plans at short notice to accommodate unexpected traditional ceremonies and other important community visitors
To use local Aboriginal interpreters and research assistant	Kinship and avoidance issues	Researchers understand and accommodate kinship and avoidance issues when working with Aboriginal people in a community
Reimbursement for time spent	Concerns of coercion or inappropriate use of given money	Food and drink after fasting
		Wave-3 Canvas bag with wrist band and water bottle with study logo, tooth brush, tooth paste and health promotion flyers
		Later, urban participants given $AUD20 voucher for retail department store

## Discussion

The Aboriginal Birth Cohort Study demonstrates that satisfactory follow-up rates of an Indigenous cohort with traditional characteristics are possible and scientific rigor can be maintained in a challenging setting. The overall vital status determined for 83% of participants and the physical examination of 72% of living participants at 20 years after recruitment are indicative of the successful retention strategies that have evolved over the course of this Indigenous population study.

These follow-up rates are comparable to other cohort studies in developed countries [15-17], which have retention techniques that were unavailable in our study. Such techniques include mail surveys, telephone surveys, birthday cards, sending mobile phone reminders and newsletters [18]. Follow-up rates of cohorts in developing populations with conditions in contrast to the ABC study are similar or higher than our study. At 3 years, a follow up rate of 83% was achieved in Vellore slum birth cohort [19] (catchment area 2.2 km^2^, population density of 17,000 per km^2^), while a Mysore birth cohort achieved 94% follow up rate at 9-10 years [20]. The Pelatos (Brazil) Birth Cohort study has a 77% follow-up rate at 23 years, achieved by visiting 98,000 households in the city and using educational data bases and telephone directories [21].

Our cohort study is consistent with other reports of hard-to-reach and marginalised populations, showing retention is often manual and labour intensive and requires flexibility, persistence and ingenuity [22]. The flexibility for this study extended to planning around weather conditions, addressing community geographic and cultural considerations and individual participant’s needs. Notable in this study is the reliance on community support and goodwill facilitated by extensive consultation and negotiation and the researchers’ flexibility, respect and transparent communication.

Developing a profile of the ABC study and the research team has been critical in establishing the legitimacy of the study and has gathered momentum with time. Continuity of field workers and particularly a male Aboriginal research team member employed over two adult data collection waves has been pivotal in engaging with shy Aboriginal men and cementing the legitimacy of the study. The inclusion of local Aboriginal research assistants remains absolutely essential to address very specific community cultural and logistic issues, arising from the large diversity in Aboriginal communities.

Consultation was sought from an institutional Aboriginal reference group which also contributed methods to engage Aboriginal communities. An ABC reference group drawn from the participants has met regularly to discuss research approaches. This small group has been invited to ‘Researcher Thank You Days’ with the local media present, and have taken part in media activities such as a television segment focussing on the ABC study.

The ABC study has been conducted within the principles of the Road Map 11 strategy, [11] including employment of local Aboriginal research assistants and training for the male research assistant. The Australian ethical guideline themes for conducting research with Indigenous people and communities have provided an ethical framework for the study with strategies evolving over time. Initial consent was given by the mothers on behalf of the participants at birth. Later at 11 years of age formal consent was again given by the mother but the child’s wishes in regard to specific procedures were respected. For adult participants, a staged consent form for data collection has been developed instead of blanket consent for all procedures (Additional file 1). This gives participants control over data collection where 10% of participants felt able to opt out of specific procedures at 18 years of age.

In view of the longitudinal nature of the study and the need for co-operation over a lifetime, individual research experiences are limited to 2 hours or less with food and drinks available after fasting bloods are taken.

This is the first documentation of practical strategies used to conduct a longitudinal study of an Indigenous birth cohort. It relates to an under-served ethnic minority group with traditional connections to land and culture and addresses the ethical guidelines for conducting research with Indigenous people. There are other reports relating to marginalised ethnic minorities [23] and minority research participants [24] but the populations described are not traditional and the retention strategies of mass mailing, emails and telephone prompts are still used in these cohorts.

The developed strategies are based on researchers experiences and have been refined over the course of the study, where two authors have been involved for 26 years and 15 years respectively. The follow-up loss of only 13% after 20 years suggests the current package of strategies is successful; however currently we are unable to disentangle the cost effectiveness of specific strategies in the package.

The strategies developed relate to diverse Indigenous populations in the northern part of Australia. Generalisation to other indigenous populations and or other Australian indigenous groups may be limited and the strategies reported may not have direct application in other settings. However some of our innovations may serve as templates for modification and encourage researchers to develop other non-conventional approaches suited to individual research settings.

The first two decades of this cohort have built significant foundations for its future. Using the approaches refined over time we are currently well placed to continue this life course study of an Indigenous birth cohort provided we maintain the same extensive consultation and negotiation processes with flexibility, respect and transparent communication.

## Conclusions

Satisfactory follow-up rates of a prospective longitudinal Indigenous birth cohort with traditional characteristics are possible while maintaining scientific rigor in a challenging setting. Approaches needed, include flexibility, respect, and transparent communication along with the adoption of culturally sensitive behaviours.

## Competing interests

The authors declare that they have no competing interests.

## Authors’ contributions

ML drove the manuscript concept, drafted the initial drafts and organised the references. SS modified draft, tables and figures and added content. GS reviewed drafts, modified tables and added content. All authors read and approved the final manuscript.

## Pre-publication history

The pre-publication history for this paper can be accessed here:

http://www.biomedcentral.com/1471-2288/14/31/prepub

## Supplementary Material

Additional file 1 Aboriginal Birth Cohort: Itemised consent form. Click here for file
