# Supplementary material for: Challenges and strategies for cohort retention and data collection in an indigenous population: Australian Aboriginal Birth Cohort
Source: BMC Med Res Methodol. 2014 Feb 26;14:31. doi: 10.1186/1471-2288-14-31 (PMC3942517; doi:10.1186/1471-2288-14-31)
Supplement: Additional file 1 — Aboriginal Birth Cohort: Itemised consent form. [file 1471-2288-14-31-S1.docx]

**Additional file 1**

**CONSENT FORM**

Aboriginal Birth Cohort Study

I, ……………………………………………………… (please print name)

*First name Last name*

consent to take part in the research project entitled: Aboriginal Birth Cohort Study

1. I acknowledge that I have read and understood the Study Information Sheet I am not aware of any medical condition which would prevent my participation, and I agree to participate in this research.
2. I have had the project fully explained to my satisfaction by the research worker. My consent is given freely.
3. I have consented to undertake the procedures as agreed to in the consent form.
4. I understand I will be asked questions in regard to health, lifestyle and behaviour and that I may choose not to answer any or all of these questions.
5. Although I understand that the purpose of this research project is to improve knowledge on young adult health it has also been explained that my involvement in this health check may not be of any direct benefit to me.
6. I have been given the opportunity to have a member of my family or a friend present while the project was explained to me.
7. I have been informed that, while information gained during the study may be published, I will not be identified and my personal results will not be divulged.
8. I understand that I am free to withdraw from the project at any time and that this will not affect medical advice in the management of my health, now or in the future.

This research project has been considered and approved by the Human Research Ethics Committee of the NT Department of Health and Menzies School of health Research.

……………………………………………………………………………………………

*(signature) (date)*

I nominate my medical results to be sent to

Name ……………………………………………………………………………….

Address………………………………………………Phone……………………...

**PROCEDURES**

**Aboriginal Birth Cohort Study**

**Name:** ………………………………………………………………………………………

**Part I I give permission for:**

**A all the assessments listed below**

*or*

**B the assessments as marked below: -**

| Measurements of height, weight, head, arm, waist, hip and body fat | yes | no |
| --- | --- | --- |
| Measurements of blood pressure | yes | no |
| Measurement of digital pulse wave | yes | no |
| Measurement of heart rate variability | yes | no |
| Measurement of lung function by the spirometer | yes | no |
|  |  |  |
| Ultrasound and measurements of kidney size | yes | no |
| Ultrasound and measurement of carotid artery wall thickness | yes | no |
| Ultrasound and measurements of thyroid size | yes | no |
|  |  |  |
| A blood sample to be taken | yes | no |
| A urine sample to be collected | yes | no |
| My blood and urine to be stored at Menzies only until sent for analysis | yes | no |
|  |  |  |
| To do “Speedy cards” on the computer | yes | no |
| **To complete a questionnaire in regards to** |  |  |
| Emotional wellbeing | yes | no |
| Health, lifestyle, education and income | yes | no |
| Alcohol and Tobacco | yes | no |
| Other Substance’s | yes | no |
|  |  |  |
| Measurement of grip strength | yes | no |
| Participate in balance test | yes | no |
| **Part II** |  |  |
| To have my picture taken and kept for records | yes | no |
| To have my picture used on poster or presentations | yes | no |
| **Part III** |  |  |
| For referral to appropriate health services if required  To have my health records checked | yes  yes | no  no |
| Allow the study to obtain information from government agencies such as education  and justice department using data linkage | yes | no |
| To be contacted in the future for a follow-up visit e.g. 4-5 years | yes | no |

……………………………………………………………………………………………

*(signature) (date)*

Name of Witness to Participants Signature (printed) ……………………………………

……………………………………………………………………………………………

*(signature) (date)*

Researcher’s Name (printed) …………………………………………

……………………………………………………………………………………………

*(signature) (date)*

Interpreter’s Name (if required, printed) …………………………………………

……………………………………………………………………………………………
